# Hyperendemic *Chlamydia trachomatis* sexually transmitted infections among females represent a high burden of asymptomatic disease and health disparity among Pacific Islanders in Fiji

**DOI:** 10.1371/journal.pntd.0008022

**Published:** 2020-01-23

**Authors:** Virginia Svigals, Alden Blair, Santha Muller, Aalisha Sahu Khan, Daniel Faktaufon, Mike Kama, Torika Tamani, Laila Esfandiari, Mollie O’Brien, Deborah Dean

**Affiliations:** 1 Center for Immunobiology and Vaccine Development, University of California San Francisco Benioff Children’s Hospital Oakland Research Institute, Oakland, California, United States of America; 2 Institute for Global Health Sciences, University of California San Francisco, San Francisco, California, United States of America; 3 Department of Health Sciences, Fiji National University College of Nursing and Health Sciences, Suva, Fiji; 4 Fiji Centre for Communicable Disease Control, Ministry of Health and Medical Services, Suva, Fiji; 5 Family Health, Ministry of Health and Medical Services, Suva, Fiji; 6 Departments of Bioengineering, University of California Berkeley and San Francisco, San Francisco, California, United States of America; 7 Departments of Medicine and Pediatrics, University of California San Francisco, San Francisco, California, United States of America; University of Liverpool, UNITED KINGDOM

## Abstract

**Background:**

*Chlamydia trachomatis* is the most common bacterial sexually transmitted infection worldwide with some of the highest prevalence rates among Pacific Island Countries where syndromic management is practiced. However, little is known about the true prevalence and risk indicators for infection among neglected populations in these countries that suffer from health disparities.

**Methodology/Principal findings:**

Consecutive sampling was used to enroll sexually active females, aged 18–40 years, attending 12 Fijian Ministry of Health and Medical Services Health Centers and outreach locations from February to December, 2018. A Behavioral Surveillance Survey was administered to assess risk indicators for infection. Signs and symptoms were recorded, and vaginal swabs were tested for *C*. *trachomatis*, *Neisseria gonorrhoeae*, *Trichomonas vaginalis*, *Candida* and bacterial vaginosis. Bivariate and multivariate logistic regression analyses were performed using R-Studio. Of 577 participants, 103 (17.85%) were infected with *C*. *trachomatis* of whom 80% were asymptomatic and only 11 met criteria for syndromic management; 38.8% of infected women were 18–24 years old with a prevalence of 30.5%. 91.7% of participants intermittently or did not use condoms. *C*. *trachomatis* infection was associated with iTaukei ethnicity (OR 21.41 [95% CI: 6.38–133.53]); two lifetime partners (OR 2.12 [95% CI: 1.08–4.18]); and *N*. *gonorrhoeae* co-infection (OR 9.56 [95% CI: 3.67–28.15]) in multivariate analyses.

**Conclusions:**

A disproportionately high burden of *C*. *trachomatis* is present among young asymptomatic women in Fiji of iTaukei ethnicity despite the low number of lifetime partners. Syndromic management and lack of barrier contraceptives contribute to hyperendemic levels. Strategic STI education and screening of at-risk adolescents, young women, and their partner(s) with appropriate treatment are urgently needed to control the epidemic.

## Author summary

Western Pacific Island Countries and Territories (PICT) represent a resource-constrained developing region that suffers from numerous health disparities. It has some of the highest rates of *Chlamydia trachomatis* sexually transmitted infections (STIs) in the world where syndromic management is practiced. Screening has been performed in only a few PICT and for specific populations such as pregnant women while little is known about risk indicators. We enrolled a diversity of women with low to high risk for acquiring *C*. *trachomatis* infection and found a high burden of asymptomatic disease at hyperendemic levels among 18–24 year old women. We also found that the highest risk for *Chlamydia* was among native Pacific Islanders of iTaukei ethnicity. Few infected women met syndromic management criteria for treatment, leaving a high number at risk of developing severe upper genital tract disease and transmitting the pathogen to their partners. STI education, testing and treatment of at-risk women and their partner(s) are urgently needed to control this epidemic. Most importantly, *C*. *trachomatis* is a risk factor for HIV acquisition and, while HIV prevalence remains low in most of the PICT, hyperendemic disease in conjunction with an economy built on tourism leaves Fiji vulnerable to the rapid spread of HIV and its consequent morbidity and mortality.

## Introduction

More than 1 million sexually transmitted infections (STIs) are acquired worldwide every day [1, 2]. Some of the highest concentrations of STIs are found among the 22 Pacific Island Countries and Territories (PICT) of the Western Pacific Ocean [3]. The majority of STIs in this region are caused by *Chlamydia trachomatis* infecting 61 million people with a prevalence of up to 44% among antenatal teens and young adults [3–5, 6]. Global estimates are as high as 131 million annual cases according to the World Health Organization (WHO) [1, 3, 7], making *C*. *trachomatis* the most common sexually transmitted bacterium worldwide.

Recent STI data from the PICT were collected in 2016 among antenatal women in Papua New Guinea. *C*. *trachomatis* prevalence was 22.9% for those 18–35 years of age; *Neisseria gonorrhoeae* was as high as 14.2% [8]. HIV and syphilis ranged from 0.8 to 1.6%. The only other PICT studies are from Samoa in 2004 and several PICT in a study from 2008 [5, 6]. These reports showed that 30.9% and 29%, respectively, of antenatal women were positive for *C*. *trachomatis* with a much higher prevalence among women under age 25 years: 44.6% in Samoa and 34% in Fiji [5, 6]. The rates for *N*. *gonorrhoeae* and syphilis were much lower than in Papua New Guinea with HIV reported at a prevalence of less than 1%.

*C*. *trachomatis* infection can present with non-specific symptoms of dysuria, vaginal or urethral discharge, lower abdominal pain, and/or dyspareunia [9]. However, approximately 80% of women and 50% of men are asymptomatic [10, 11], creating a challenge for infection control and treatment. This is complicated by the fact that the WHO recommends syndromic management, which relies primarily on signs and symptoms, in low-income and low-resource settings [12]. Undiagnosed and therefore untreated *C*. *trachomatis* can lead to pelvic inflammatory disease, ectopic pregnancy, chronic pelvic pain, infertility and adverse pregnancy outcomes in addition to an increased risk of cervical cancer and HIV [9, 13, 14, 15].

The Fijian Ministry of Health and Medical Services (MoHMS) and WHO determined in 2018 that 70% of all diagnosed STIs were identified in adolescents and young adults [1]. High rates of unprotected sex and lack of STI surveillance, especially for *C*. *trachomatis* and *N*. *gonorrhoeae*, were the main barriers facing STI management [1, 16–18]. The lack of screening is attributed to the high costs and unavailability of certified diagnostic tests, which is complicated by supply chain logistics, few clinical laboratories to run the tests and lack of a reliable reporting system [5, 17, 19]. These barriers to screening are consistent with other low-resource settings.

**Fig 1:**
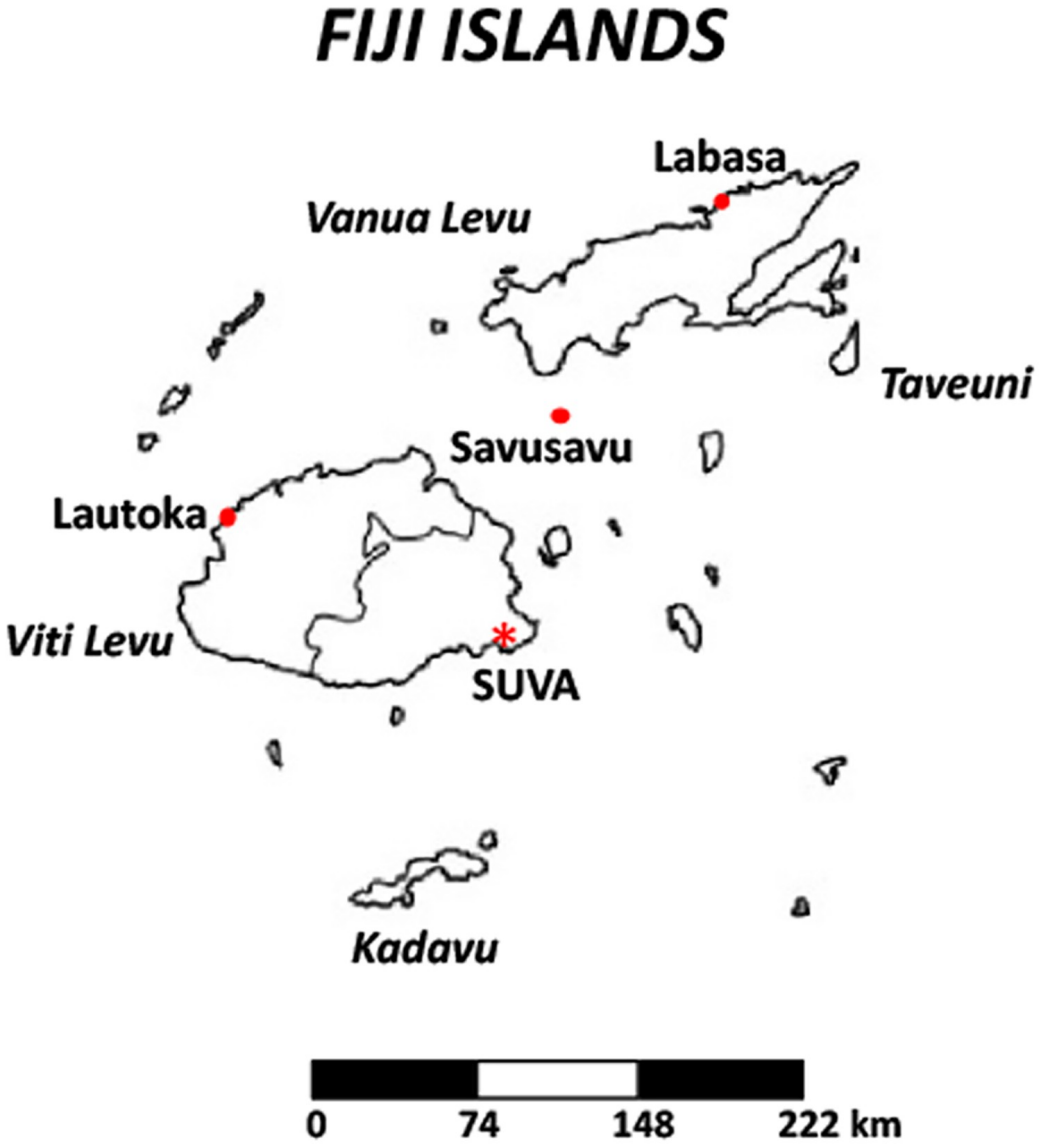
Map of the archipelago of Fiji showing the main island of Viti Levu, including the capital city of Suva. The Eastern half of Viti Levu comprises what is known as the Central Division.

Given the lack of data on *C*. *trachomatis* infections and risk indicators among non-pregnant adolescent and adult women in Fiji and the historically high rates among antenatal women from the study in 2008 [5], we consecutively enrolled women attending MoHMS Health Centers to capture a diversity of low to high risk women residing in the Central Division of Viti Levu, Fiji, the most populated region of the country (Fig 1). Current prevalence and risk indicators for *C*. *trachomatis* STIs were determined, providing insight into the demographic and socio-behavioral factors that drive *C*. *trachomatis* infection in Fiji—knowledge that is critical for designing optimal control strategies.

## Methods

### Ethics statement

Institutional Review Boards of UCSF Benioff Children’s Hospital Oakland Research Institute and the Fijian MoH&MS approved this study in accordance with the Declaration of Helsinki. Written informed consent was provided by all participants. Data were de-identified for the analyses presented here.

### Study design, population, sample size and definitions

This cross-sectional study used a non-probability, consecutive sampling method to enroll women aged 18 to 40 years from February 2018 to December 2018. Enrollment sites included 12 MoH&MS Health Centers and outreach locations in the Central Division of Viti Levu where the capital Suva is located (Fig 1). Urban outreach included SAN Fiji sex workers and military women. Rural catchment included clinics in Cautata, Ucunivanua and Sote. All women were invited to attend. Participation was entirely voluntary.

Women were excluded from the study if they had been treated with antibiotics in the prior month, had a diagnosis of cancer, had untreated syphilis, or were pregnant. The latter three exclusion criteria were used to avoid potential confounding from an increased risk of infection associated with an immunocompromised condition. Women were consented and enrolled if they were within the specified age range, sexually active, and willing to be examined and provide clinical samples for the study.

A sample size of 500 women was estimated to generate adequate precision for estimates of prevalence with a power of at least 80% to detect reasonably small differences between outcome proportions for groups of participants distinguished by the characteristics of interest.

Transactional sex was defined as acceptance of money for sex. STI symptoms included vaginal discharge, unusual or foul-smelling vaginal discharge, dyspareunia, bleeding or spotting during or after sex not associated with menses, and/or lower abdominal pain. Cervicitis was defined as purulent discharge in the endocervical canal (or on the swab) and/or sustained bleeding when a swab was inserted through the cervical os according to U.S. Centers for Disease Control and Prevention guidelines [11]. Clinical bacterial vaginosis (BV) was diagnosed based on three or more Amsel criteria [20]: homogeneous vaginal discharge, >20% clue cells on wet prep, vaginal pH ≥4.5, and fishy amine odor when KOH was applied to vaginal material on a glass slide.

### Survey data

Socio-demographic and behavioral data were obtained through a survey adapted from the Family Health International HIV/AIDs/STD Behavioral Surveillance Study (BSS) for Adults [18, 21] and following behavioral surveillance guidelines outlined by Family Health International and U.S. Agency for International Development [22]. Questions assessing sexual coercion and alcohol use were adapted from the Sexual Coercion in Intimate Relationships Scale (SCIRS) and the AUDIT-C Questionnaire [23, 24]. The sexual coercion score measures frequency and severity of physical sexual violence as well as psychological and behavioral tactics, including attempts to discourage or thwart condom use and verbal threats to withhold resources, terminate the relationship, or solicit sex from others should sexual access be denied. Alcohol scoring was based on frequency of drinking episodes in the past year and typical number of drinks consumed on a single occasion; heavy drinking was defined as six or more drinks on a single occasion. Minor modifications were made for cultural security, time feasibility, and inclusion of study-specific indicators.

Adapted survey questions were categorized into nine high-risk indicators to measure behaviors that may directly influence *C*. *trachomatis* acquisition and transmission. These indicators included: age, multiple sex partners, unprotected sex with a partner and/or transactional partner, concurrent partnerships, partner STI status, incorrect beliefs about STI transmission and prevention methods, sexual coercion, and alcohol use. Lifetime number of sex partners, and number in the past year and month, included any sex partner regardless of type of sexual experience or partner’s gender.

Participants self-administered the survey in a private room; assistance with questions was available as needed. Participants were evaluated for symptoms and signs of STIs, and underwent pelvic and rectal examinations to discern any gross pathology, evidence of pain/discomfort or hematochezia.

### Sample collection and laboratory procedures

Trained clinicians collected vaginal samples by inserting a swab (FLOQswab, Copan, Murietta, CA) halfway between the introitus and cervix and rotating four times to saturate the swab. One swab was immediately placed in transport media (SWAB/A-50 Collection Kit, Cepheid, Sunnyvale, CA) for *C*. *trachomatis* and *N*. *gonorrhoeae* testing, stored at 4°C until same-day transport to the Fiji Communicable Disease Laboratory, and processed using the Xpert CT/NG Assay (Cepheid) according to manufacturer’s instructions. All kits were provided by the study.

A second swab collected similarly was touched to a 0.0–10 scale pH strip (Merck, Kenilworth, NY) and then rolled 180° onto two glass slides: 10% KOH was applied to one slide to detect *Candida* and normal saline was applied to the other slide to detect clue cells and *Trichomonas vaginalis* (*Tv*), and read within 30 minutes using standard techniques [25]. The percent of clue cells were noted. One or more motile trichomonads and pseudohyphae/budding yeast were considered presumptive of *Tv* and *Candida*, respectively.

### Analysis

Data were analyzed using R version 3.5.0 [26]. Participant demographics and behaviors were first examined via univariate summaries including proportions and frequency tables. Associations were explored between a positive *C*. *trachomatis* test and population characteristics including socio-economic indicators, sexual behaviors, alcohol use, sexual coercion scores[23, 24], STI symptoms, clinical signs and comorbid STIs, BV and *Candida*.

A descriptive multivariable logistic regression model was built to explore which factors were associated with *C*. *trachomatis* after accounting for potential confounding. Variables were included in the model based on their significance from prior epidemiologic literature and the bivariate analyses to account for cohort-specific variations. The latter variables were included in the final model with empirically-significant variables based on the model’s Bayesian Information Criterion (BIC) score.

## Results

From February to December, 2018, 577 participants were enrolled and completed the survey along with sampling for STIs. These women attended Health Centers/Outreach clinics for a variety of reasons including annual pap test, family planning, contraception, general checkup, infertility or concerns about having an STI. Table 1 includes participant characteristics and univariate summaries by relevant variables including raw numbers and percentages. The median age for participants was 30 years. The majority of women identified as iTaukei (n = 345[59.8%]) followed by Indo-Fijian (n = 156 [27.0%]) with the remainder divided across eight other groups. Over half had a university-level degree, and half were fully and/or self-employed, followed by homemakers (n = 104[18.0%]) and fulltime students (n = 77[13.3%]). Over 60% were married and approximately 20% were single. Five (0.87%) reported transactional sex.

**Table 1:** Baseline characteristics and bivariate analyses of associations with *C*. *trachomatis* infection.

		***C*. *trachomatis* Results**				
	**Total**	**Negative**	**Positive**	***P*-value**	**OR**	**95% CI**
	**(n = 577)**	**(n = 474)**	**(n = 103)**			
**Age (median, IQR)**	30 (25–34)	30 (26–35)	26 (22.5–31)	<0.0001	**-**	**-**
**Age Group**						
18–24 years	131 (22.7%)	91 (19.2%)	40 (38.8%)	<0.0001	0	ref
25–30 years	188 (32.6%)	152 (32.1%)	36 (35.0%)		0.54	0.32 to 0.91
30–40 years	258 (33.7%)	231 (48.7%)	27 (26.2%)		0.27	0.15 to 0.46
**Health Center or Outreach site**						
Women’s Wellness HC	268 (46.4%)	213 (44.9%)	55 (53.4%)	0.61	0	ref
Makoi HC	11 (1.9%)	8 (1.7%)	3 (2.9%)		1.45	0.31 to 5.21
Nausori HC	36 (6.2%)	31 (6.5%)	5 (4.9%)		0.62	0.21 to 1.55
Valelevu HC	94 (16.3%)	82 (17.3%)	12 (11.7%)		0.57	0.28 to 1.08
Reproductive HC	30 (5.2%)	23 (4.9%)	7 (6.8%)		1.18	0.45 to 2.76
University clinic #1	40 (6.9%)	32 (6.8%)	8 (7.8%)		0.97	0.37 to 2.13
University clinic #2	50 (8.7%)	44 (9.3%)	6 (5.8%)		0.53	0.19 to 1.22
Outreach—Rural	21 (3.6%)	18 (3.8%)	3 (2.9%)		0.65	0.15 to 1.99
Outreach—Urban	27 (4.7%)	23 (4.9%)	4 (3.9%)		0.67	0.19 to 1.94
**Ethnicity**						
European	6 (1.0%)	6 (1.3%)	0 (0.0%)	< 0.0001	0	-
Indo-Fijian	156 (27.0%)	154 (32.5%)	2 (1.9%)		0	ref
iTaukei Fijian	345 (59.8%)	258 (54.4%)	87 (84.5%)		25.97	8.05 to 159.06
Lauan	3 (0.5%)	3 (0.6%)	0 (0.0%)		0	-
Rambian	4 (0.7%)	3 (0.6%)	1 (1.0%)		25.67	1.03 to 360.61
Rotuman	14 (2.4%)	12 (2.5%)	2 (1.9%)		12.83	1.44 to 115.05
Other Pacific Islanders	17 (2.9%)	15 (3.2%)	2 (1.9%)		10.27	1.17 to 90.76
Other Caucasian	4 (0.7%)	4 (0.8%)	0 (0.0%)		0	-
Other	22 (3.8%)	18 (3.8%)	4 (3.9%)		17.11	3.12 to 129.88
		***C*. *trachomatis* Results**				
	**Total**	**Negative**	**Positive**	***P*-value**	**OR**	**95% CI**
	**(n = 577)**	**(n = 474)**	**(n = 103)**			
No answer	6 (1.0%)	1 (0.2%)	5 (4.9%)		385	41.28 to 978.24
**Education level**						
Primary School (Class 1–8)	17 (2.9%)	12 (2.5%)	5 (4.9%)	0.47	0	ref
Secondary School (Form 3–7)	191 (33.1%)	156 (32.9%)	35 (34.0%)		0.54	0.19 to 1.78
University	320 (55.5%)	264 (55.7%)	56 (54.4%)		0.51	0.18 to 1.65
Post-University (Graduate or Doctorate)	46 (8.0%)	41 (8.6%)	5 (4.9%)		0.29	0.07 to 1.21
Never Attended School	1 (0.2%)	1 (0.2%)	0 (0.0%)		0	-
Missing	2 (0.3%)	0 (0.0%)	2 (1.9%)		-	-
**Employment status**						
Employed full-time or Self-employed	289 (50.1%)	247 (52.1%)	42 (40.8%)	0.019	0	ref
Employed part-time	36 (6.2%)	28 (5.9%)	8 (7.8%)		1.68	0.68 to 3.79
Homemaker/Domestic duties	104 (18.0%)	90 (19.0%)	14 (13.6%)		0.91	0.46 to 1.72
Full-time Student	77 (13.3%)	58 (12.2%)	19 (18.4%)		1.93	1.03 to 3.52
Retired	0 (0.0%)	0 (0.0%)	0 (0.0%)		-	-
Unemployed	53 (9.2%)	37 (7.8%)	16 (15.5%)		2.54	1.28 to 4.92
Other	9 (1.6%)	9 (1.9%)	0 (0.0%)		0	-
Missing	9 (1.6%)	5 (1.1%)	4 (3.9%)		-	-
**Marital status**						
Married	371 (64.3%)	318 (67.1%)	53 (51.5%)	0.002	0	ref
Single	114 (19.8%)	81 (17.1%)	33 (32.0%)		2.44	1.48 to 4.01
Partner relationship but not married	73 (12.7%)	60 (12.7%)	13 (12.6%)		1.3	0.64 to 2.47
Divorced or separated	15 (2.6%)	14 (3.0%)	1 (1.0%)		0.43	0.02 to 2.20
Widowed	1 (0.2%)	1 (0.2%)	0 (0.0%)		0	-
Other	1 (0.2%)	0 (0.0%)	1 (1.0%)		0	-
Missing	2 (0.3%)	0 (0.0%)	2 (1.9%)		-	-
**Problematic alcohol use**						
3+	327 (56.7%)	257 (54.2%)	70 (68.0%)	0.012	0	ref
		***C*. *trachomatis* Results**				
	**Total**	**Negative**	**Positive**	***P*-value**	**OR**	**95% CI**
	**(n = 577)**	**(n = 474)**	**(n = 103)**			
Below 3	250 (43.3%)	217 (45.8%)	33 (32.0%)		0.56	0.35 to 0.87
**Knows one can have STI without symptoms**						
No	143 (24.8%)	113 (23.8%)	30 (29.1%)	0.2	0	ref
Yes	411 (71.2%)	344 (72.6%)	67 (65.0%)		0.73	0.46 to 1.20
Missing	23 (4.0%)	17 (3.6%)	6 (5.8%)		-	-
**Knows diseases can be transmitted by sex**					
No	132 (22.9%)	111 (23.4%)	21 (20.4%)	0.52	0	ref
Yes	424 (73.5%)	345 (72.8%)	79 (76.7%)		1.21	0.73 to 2.09
Missing	21 (3.6%)	18 (3.8%)	3 (2.9%)		-	-
**Has heard of condoms**						
No	31 (5.4%)	23 (4.9%)	8 (7.8%)	0.23	0	ref
Yes	531 (92.0%)	438 (92.4%)	93 (90.3%)		0.61	0.28 to 1.49
Missing	15 (2.6%)	13 (2.7%)	2 (1.9%)		-	-
**Knows condoms decreases risk of STIs**						
No	46 (8.0%)	36 (7.6%)	10 (9.7%)	0.55	0	ref
Yes	523 (90.6%)	431 (90.9%)	92 (89.3%)		0.77	0.38 to 1.69
Missing	8 (1.4%)	7 (1.5%)	1 (1.0%)		-	-
**Knows where to obtain free condoms**						
No	69 (12.0%)	54 (11.4%)	15 (14.6%)	0.4	0	ref
Yes	497 (86.1%)	410 (86.5%)	87 (84.5%)		0.76	0.42 to 1.46
Missing	11 (1.9%)	10 (2.1%)	1 (1.0%)		-	-
**Condom use–regular partner**						
always	38 (7.5)	35 (8.4%)	3 (3.5%)	0.008	0	ref
sometimes	140 (27.8%)	125 (29.9%)	15 (17.4%)		2.42	1.10 to 5.05
never	326 (64.7%)	258 (61.7%)	68 (79.1%)		3.07	1.07 to 13.03
**Condom use–casual partner**						
always	13 (20.0%)	12 (24.0%)	1 (6.7%)	0.18	0	ref
		***C*. *trachomatis* Results**				
	**Total**	**Negative**	**Positive**	***P*-value**	**OR**	**95% CI**
	**(n = 577)**	**(n = 474)**	**(n = 103)**			
sometimes	50 (76.9%)	37 (74.0%)	13 (86.6%)		4.21	0.72 to 80.58
never	2 (3.1%)	1 (2.0%)	1 (6.7%)		3.07	1.07 to 13.03
**Age of sexual debut**						
Mean (SD)	20.2 (±2.9)	20.4 (±3.1)	19.4 (±2.2)	0.006	0.89	0.82 to 0.96
Missing	25 (4.3%)	25 (5.3%)	0 (0%)		-	-
**Lifetime number of sexual partners**						
1	227 (39.3%)	200 (42.2%)	27 (26.2%)	0.001	0	ref
2	125 (21.7%)	95 (20.0%)	30 (29.1%)		2.34	1.32 to 4.17
3–6	139 (24.1%)	108 (22.8%)	31 (30.1%)		2.13	1.21 to 3.77
7–10	34 (5.9%)	24 (5.1%)	10 (9.7%)		3.09	1.29 to 7.03
10+	18 (3.1%)	17 (3.6%)	1 (1.0%)		0.44	0.03 to 2.26
Invalid response	1 (0.2%)	0 (0.0%)	1 (1.0%)		0	-
Missing	33 (5.7%)	30 (6.3%)	3 (2.9%)		-	-
**Yearly number of sexual partners**						
0	11 (1.9%)	10 (2.1%)	1 (1.0%)	0.57	0.49	0.15 to 0.26
1	412 (71.4%)	342 (72.2%)	70 (68.0%)		0	ref
2	58 (10.1%)	45 (9.5%)	13 (12.6%)		1.41	0.70 to 2.69
3–6	41 (7.1%)	30 (6.3%)	11 (10.7%)		1.79	0.82 to 3.65
7–10	8 (1.4%)	7 (1.5%)	1 (1.0%)		0.7	0.04 to 4.00
10+	3 (0.5%)	2 (0.4%)	1 (1.0%)		2.44	0.11 to 25.84
Can’t remember	1 (0.2%)	1 (0.2%)	0 (0.0%)		0	-
Missing	43 (7.5%)	37 (7.8%)	6 (5.8%)		-	-
**Monthly number of sexual partners**						
0	53 (9.2%)	41 (8.6%)	12 (11.7%)	0.005	1.46	0.71 to 2.83
1	462 (80.1%)	385 (81.2%)	77 (74.8%)		0	ref
2	18 (3.1%)	14 (3.0%)	4 (3.9%)		1.42	0.40 to 4.11
3	2 (0.3%)	1 (0.2%)	1 (1.0%)		5	0.20 to 127.35
4	3 (0.5%)	0 (0.0%)	3 (2.9%)		0	-
		***C*. *trachomatis* Results**				
	**Total**	**Negative**	**Positive**	***P*-value**	**OR**	**95% CI**
	**(n = 577)**	**(n = 474)**	**(n = 103)**			
10	1 (0.2%)	0 (0.0%)	1 (1.0%)		0	-
Missing	38 (6.6%)	33 (7.0%)	5 (4.9%)		-	-
**Sex of sexual partner**						
Male	535 (92.7%)	442 (93.2%)	93 (90.3%)	0.11	0	ref
Female	16 (2.8%)	10 (2.1%)	6 (5.8%)		2.85	0.95 to 7.88
Transgender/transsexual M—>F	8 (1.4%)	8 (1.7%)	0 (0.0%)		0	-
Transgender/transsexual F—>M	6 (1.0%)	5 (1.1%)	1 (1.0%)		0.95	0.05 to 5.98
Missing	12 (2.1%)	9 (1.9%)	3 (2.9%)		-	-
**Has sex with a regular partner**						
No	50 (8.7%)	39 (8.2%)	11 (10.7%)	0.44	0	ref
Yes	522 (90.5%)	432 (91.1%)	90 (87.4%)		0.74	0.38 to 1.56
Missing	5 (0.9%)	3 (0.6%)	2 (1.9%)		-	-
**Regular partner has sex with others**						
Yes	28 (37.3%)	20 (36.4%)	8 (40.0%)	0.0003	0	ref
No	16 (21.3%)	16 (29.1%)	0 (0.0%)		0.41	0.22 to 0.80
I don’t know	12 (16.0%)	4 (7.3%)	8 (40.0%)		0.95	0.47 to 1.95
Missing	19 (25.3%)	15 (27.3%)	4 (20.0%)		-	-
**Has sex with a casual partner**						
No	457 (79.2%)	380 (80.2%)	77 (74.8%)	0.052	0	ref
Yes	75 (13.0%)	55 (11.6%)	20 (19.4%)		1.79	1.00 to 3.12
Missing	45 (7.8%)	39 (8.2%)	6 (5.8%)		-	-
**Engage in vaginal sex**						
No	35 (6.1%)	28 (5.9%)	7 (6.8%)	0.65	0	ref
Yes	496 (86.0%)	409 (86.3%)	87 (84.5%)		0.85	0.38 to 2.17
Missing	46 (8.0%)	37 (7.8%)	9 (8.7%)			
**Engage in anal sex**						
Yes	98 (17.0%)	79 (16.7%)	19 (18.4%)	0.41	0	ref
No	409 (70.9%)	340 (71.7%)	69 (67.0%)		0.84	0.49 to 1.52
		***C*. *trachomatis* Results**				
	**Total**	**Negative**	**Positive**	***P*-value**	**OR**	**95% CI**
	**(n = 577)**	**(n = 474)**	**(n = 103)**			
I don’t know	23 (4.0%)	17 (3.6%)	6 (5.8%)		1.47	0.48 to 4.08
Missing	47 (8.1%)	38 (8.0%)	9 (8.7%)		-	-
**Sexual coercion score**						
Over 7	445 (77.1%)	363 (76.6%)	82 (79.6%)	0.6	0	ref
Under 7	132 (22.9%)	111 (23.4%)	21 (20.4%)		0.84	0.49 to 1.39
**Has ever had an STI**						
No	491 (85.1%)	400 (84.4%)	91 (88.3%)	0.26	0	ref
Yes	57 (9.9%)	50 (10.5%)	7 (6.8%)		0.62	0.25 to 1.32
I don’t know	17 (2.9%)	16 (3.4%)	1 (1.0%)		0.27	0.02 to 1.37
Missing	12 (2.1%)	8 (1.7%)	4 (3.9%)		-	-
**Sexual partner has ever had an STI**						
Yes	35 (6.1%)	30 (6.3%)	5 (4.9%)	0.066	0	ref
No	415 (71.9%)	350 (73.8%)	65 (63.1%)		1.11	0.45 to 3.36
I don’t know	116 (20.1%)	87 (18.4%)	29 (28.2%)		2	0.76 to 6.29
Missing	11 (1.9%)	7 (1.5%)	4 (3.9%)		-	-

**IQR**, Interquartile Range; **OR**, Odds Ratio; **95% CI**, 95% Confidence Interval; **Other**, mixed Pacific Islanders with other ethnicities.

Table 1 also presents the bivariate associations with *C*. *trachomatis*, including *P*-values, odds ratios (OR), and 95% confidence intervals (95% CI). A total of 103 (17.85%) women tested positive for *C*. *trachomatis*; one (20%) of five women who reported transactional sex was positive. Women with *C*. *trachomatis* had a median age four years younger than those who were not positive (26 vs 30, *P*<0.0001). Those 18–24 years of age were more likely to have *C*. *trachomatis* infection (38.8%; *P*<0.0001).

Compared to women identifying as Indo-Fijian, all other ethnic groups had higher odds of testing positive for *C*. *trachomatis* with iTaukei Fijians being 25.97 times more likely to test positive (95% CI, 8.05–159.06) (Table 1). Women who were fulltime students (OR 1.93, 95% CI 1.03–3.52) or unemployed (OR 2.54, 95% CI 1.28–4.92) were more likely to test positive than those who were fully employed, as were single women compared to married women (OR 2.44, 95% CI 1.48–4.01). Certain sexual risk factors were also associated with *C*. *trachomatis* positivity, including two to 10 lifetime sexual partners (*P* = 0.001) and a trend for sex with a casual partner (*P* = 0.052; 95% CI 1.00–3.12). Lack of condom use was greater among women with *C*. *trachomatis* who have sex with a regular partner compared to women without *C*. *trachomatis* (*P*<0.008; 95% CI 1.07–13.03) despite the high level of knowledge about the importance of condom use. Overall, 542 (91.7%) participants did not use or intermittently used condoms.

Table 2 shows the association of other STIs, BV and *Candida* with *C*. *trachomatis* infection. While there were no associations with *Tv*, BV or *Candida*, participants with *N*. *gonorrhoeae* were significantly more likely to have *C*. *trachomatis* (*P*<0.0001; 95% CI 7.36–44.56).

**Table 2:** Bivariate analyses of other STIs, bacterial vaginosis and candida associations with C. trachomatis infection.

		*C*. *trachomatis* Results				
	Total	Negative	Positive	*P*-value	OR	95%CI
	(n = 577)	(n = 474)	(n = 103)			
***Trichomonas vaginosis***						
Negative	479 (83.0%)	397 (83.8%)	82 (79.6%)	0.45	0	ref
Positive	51 (8.8%)	39 (8.2%)	12 (11.7%)		0.67	0.35 to 1.39
Not done	46 (8.0%)	37 (7.8%)	9 (8.7%)		0.79	0.29 to 2.09
Missing	1 (0.2%)	1 (0.2%)	0 (0.0%)		-	-
***Neisseria gonorrhoeae***						
Negative	549 (95.1%)	467 (98.5%)	82 (79.6%)	< 0.0001	0	ref
Positive	28 (4.9%)	7 (1.5%)	21 (20.4%)		17.09	7.36 to 44.56
**Bacterial vaginosis**						
Negative	377 (65.3%)	316 (66.7%)	61 (59.2%)	0.31	0	ref
Positive	153 (26.5%)	120 (25.3%)	33 (32.0%)		0.7	0.44 to 1.14
Not done	46 (8.0%)	37 (7.8%)	9 (8.7%)		0.88	0.37 to 1.95
Missing	1 (0.2%)	1 (0.2%)	0 (0.0%)		-	-
***Candida***						
Negative	459 (71.6%)	373 (78.6%)	86 (83.5%)	0.22	0	ref
Positive	74 (12.8%)	66 (13.9%)	8 (7.8%)		1.9	0.88 to 4.11
Missing	44 (7.6%)	34 (7.2%)	9 (8.7%)		-	-

**OR**, Odds Ratio; **95% CI**, 95% Confidence Interval.

There were no associations between *C*. *trachomatis* positivity and symptoms (Table 3). However, signs of cervical discharge (*P* = 0.0005; 95% CI 1.44–3.66), cervicitis (*P* = 0.02; 95% CI 1.12–3.00), and cervical motion tenderness (*P* = 0.032; 95% CI 1.10–5.05) were associated with *C*. *trachomatis* (Table 3). Only 11 (10.7%) *C*. *trachomatis* infected participants met the criteria for syndromic management [12].

**Table 3:** Non-specific symptoms and clinical signs associated with *C*. *trachomatis* infection.

		***C*. *trachomatis* results**				
	**Total**	**Negative**	**Positive**	***P*-value**	**OR**	**95%CI**
	**(n = 577)**	**(n = 474)**	**(n = 103)**			
**Non-specific STI symptoms**						
**Dysuria**						
No	509 (88.2%)	422 (89.0%)	87 (84.5%)	0.48	0	ref
Yes	62 (10.7%)	49 (10.3%)	13 (12.6%)		1.29	0.65 to 2.41
Missing	6 (1.0%)	3 (0.6%)	3 (2.9%)		-	-
**Lower abdominal pain**						
No	389 (67.4%)	327 (69.0%)	62 (60.2%)	0.16	0	ref
Yes	183 (31.7%)	145 (30.6%)	38 (36.9%)		1.38	0.88 to 2.16
Missing	5 (0.9%)	2 (0.4%)	3 (2.9%)		-	-
**Cramping**						
No	531 (92.0%)	440 (92.8%)	91 (88.3%)	0.83	0	ref
Yes	39 (6.8%)	32 (6.8%)	7 (6.8%)		1.06	0.42 to 2.34
Missing	7 (1.2%)	2 (0.4%)	5 (4.9%)		-	-
**Dyspareunia**						
No	466 (80.8%)	388 (81.9%)	78 (75.7%)	0.26	0	ref
Yes	103 (17.9%)	81 (17.1%)	22 (21.4%)		1.35	0.78 to 2.27
Missing	8 (1.4%)	5 (1.1%)	3 (2.9%)		-	-
**Bleeding/spotting after intercourse**						
No	536 (92.9%)	444 (93.7%)	92 (89.3%)	0.49	0	ref
Yes	36 (6.2%)	28 (5.9%)	8 (7.8%)		1.38	0.57 to 2.99
Missing	5 (0.9%)	2 (0.4%)	3 (2.9%)		-	-
**Vaginal itching**						
No	438 (75.9%)	363 (76.6%)	75 (72.8%)	0.7	0	ref
Yes	133 (23.1%)	108 (22.8%)	25 (24.3%)		1.12	0.67 to 1.83
Missing	6 (1.0%)	3 (0.6%)	3 (2.9%)		-	-
**Vaginal discharge**						
No	416 (72.1%)	345 (72.8%)	71 (68.9%)	0.71	0	ref
		***C*. *trachomatis* results**				
	**Total**	**Negative**	**Positive**	***P*-value**	**OR**	**95%CI**
	**(n = 577)**	**(n = 474)**	**(n = 103)**			
Yes	155 (26.9%)	126 (26.6%)	29 (28.2%)		1.12	0.69 to 1.79
Missing	6 (1.0%)	3 (0.6%)	3 (2.9%)		-	-
**Clinical signs**						
**Cervical discharge**						
No	403 (69.8%)	348 (73.4%)	55 (53.4%)	0.0005	0	ref
Yes	146 (25.3%)	107 (22.6%)	39 (37.9%)		2.31	1.44 to 3.66
Missing	28 (4.9%)	19 (4.0%)	9 (8.7%)		-	-
**Cervical effacement**						
No	409 (70.9%)	342 (72.2%)	67 (65.0%)	0.41	0	ref
Yes	120 (20.8%)	96 (20.3%)	24 (23.3%)		1.28	0.75 to 2.12
Missing	48 (8.3%)	36 (7.6%)	12 (11.7%)		-	-
**Cervicitis**						
No	423 (73.3%)	359 (75.7%)	64 (62.1%)	0.02	0	ref
Yes	121 (21.0%)	91 (19.2%)	30 (29.1%)		1.85	1.12 to 3.00
Missing	33 (5.7%)	24 (5.1%)	9 (8.7%)		-	-
**Cervical motion tenderness**						
No	515 (89.3%)	430 (90.7%)	85 (82.5%)	0.032	0	ref
Yes	34 (5.9%)	23 (4.9%)	11 (10.7%)		2.42	1.10 to 5.05
Missing	28 (4.9%)	21 (4.4%)	7 (6.8%)		-	-

**OR**, Odds Ratio; **95% CI**, 95% Confidence Interval.

Table 4 reports the results of the multivariable model of best fit including unadjusted odds ratios (UOR), adjusted odds ratios (AOR) and associated 95% CI. The final model included ethnicity, which, due to the large number of small sub-groups, was condensed to Indo-Fijian, iTaukei Fijian and Other (primarily other Pacific Islanders), marital status, AUDIT-C score, lifetime sexual partners, scoring above seven on the sexual coercion score, and *N*. *gonorrhoeae* infection. Both iTaukei (AOR 21.41, 95% CI 6.38–133.53) and Other ethnicities (AOR 10.54, 95% CI 2.50–72.56) were more likely to test positive for *C*. *trachomatis* as were women with two lifetime partners (AOR 2.12, 95% CI 1.08–4.18). There were no associations between *C*. *trachomatis* and marital status, alcohol use, or Sexual Coercion Score. Women with *N*. *gonorrhoeae* were 9.56 times more likely to have *C*. *trachomatis* (95% CI 3.67–28.15).

**Table 4:** Multivariate model for variables associated with *C*. *trachomatis* infection.

Variable	UOR	95% CI	AOR	95% CI
**Age Group**				
18–25 years	0	ref	0	ref
25–30 years	0.54	0.32 to 0.91	0.59	0.30 to 1.19
30–40 years	0.27	0.15 to 0.46	0.29	0.13 to 0.60
**Ethnicity**				
Indo-Fijian	0	ref	0	ref
iTaukei Fijian	25.97	8.05 to 159.06	21.41	6.38 to 133.53
Other	17.39	4.68 to 112.82	10.54	2.50 to 72.56
**Marital Status**				
Married	0	ref	0	ref
Single	2.44	1.48 to 4.01	0.86	0.42 to 1.72
Partner relationship, not married	1.3	0.64 to 2.47	0.4	0.17 to 0.91
Divorced or separated	0.43	0.02 to 2.20	0.39	0.02 to 2.40
Widowed	0	-	0	-
Other	0	-	0	-
**Alcohol use score**				
3 or more	0	ref	0	ref
Below 3	0.56	0.35 to 0.87	0.72	0.41 to 1.24
**Lifetime number of sexual partners**				
1	0	ref	0	ref
2	2.34	1.32 to 4.17	2.12	1.08 to 4.18
3–6	2.13	1.21 to 3.77	1.61	0.83 to 3.14
7–10	3.09	1.29 to 7.03	2.18	0.77 to 5.96
10+	0.44	0.02 to 2.26	0.28	0.01 to 2.01
Invalid response	0	-	0	-
**Sexual Coercion Score**				
7 or more	0	ref	0	ref
under 7	0.84	0.49 to 1.39	1.12	0.58 to 2.09
***Neisseria gonorrhoeae***				
No	0	ref	0	ref
Yes	17.09	7.36 to 44.56	9.56	3.67 to 28.15

**UOR**, Unadjusted Odds Ratio; **AOR**, Adjusted Odds Ratio; **95% CI**, 95% Confidence Interval; **Other**, other Pacific Islanders, European, Caucasian and mixed Pacific Islanders with other ethnicities.

## Discussion

Our population comprised Fijian women who were seeking health care for various reasons not restricted to infertility or STI screening. In total, 17.85% were infected with *C*. *trachomatis*, which far exceeds the WHO estimated 4.2% [7] global prevalence of *C*. *trachomatis* among women aged 15–49 years, although these data are from 2012. Non-pregnant women under age 25 had the highest prevalence (30.5%) consistent with reports from over a decade ago where 29%-34% of antenatal women were infected [5], indicating an ongoing epidemic of *C*. *trachomatis* STIs. Our data are also similar to a study of females engaging in transactional sex in Vanuatu in 2011 (36%) [27] but surprisingly higher than a 2014 study of female transactional sex workers in Fiji where 26% of the women in a similar age group were infected [28]. However, this lower prevalence in the latter study may reflect the fact that only urine was used for screening. Previous research has shown that urine testing will miss up to 30% of endocervical *C*. *trachomatis* infections [19, 29]. Interestingly, in the only published study from the PICT of non-pregnant females, urine testing showed a prevalence of 36% among women 18 to 29 years of age in Samoa [30], suggesting that genital prevalence may be even higher.

A number of risk factors for *C*. *trachomatis* infection were identified that are similar to other studies [31]. These included age less than 25 years, single marital status, and more than one partner. While no association was seen between *C*. *trachomatis* and problematic alcohol use, as defined by AUDIT-C, the high level of consumption with over 56% of all participants and 68% of *C*. *trachomatis* positive participants screening for potentially hazardous drinking is worrisome.

For comorbid infections, the majority of women with *N*. *gonorrhoeae* were co-infected with *C*. *trachomatis*, which is consistent with other studies [32, 33]. Despite the high numbers of women with BV (153 [29%] of 530 tested), the prevalence of *C*. *trachomatis* positive women with BV was not significantly different from those without BV, although BV is a known risk factor for *C*. *trachomatis* STIs [34]. These data suggest that the high prevalence of both *C*. *trachomatis* and BV in the population obscure the ability to discern risk associations.

Previous studies in the PICT have not evaluated ethnicity as a risk factor for *C*. *trachomatis* infection. Here, we found that women of iTaukei ethnicity were 21.41 times more likely to have *C*. *trachomatis* infection. While over twice as many iTaukei were seen in our study compared to Indo-Fijians (345 vs 156), 84.5% of iTaukei were infected compared to only 1.9% of Indo-Fijians (*P*<0.0001). This contrasts with the transactional sex worker study where Indo-Fijians had a much higher prevalence of *C*. *trachomatis* infection (29% vs 20%) [28], although this is not surprising given the overall high exposure to STIs. The majority of the participants in our study were not transactional sex workers, and both ethnic groups were seen in all Health Centers and Outreach clinics. There was also no difference in iTaukei and Indo-Fijian ethnicity or risk for *C*. *trachomatis* by clinic. However, the highest prevalence of *C*. *trachomatis* was found at the Makoi Health Center (27%) that serves individuals of lower socio-economic status, the Reproductive Health Clinic (23%) that serves patients at high risk for STIs, and the University clinic #1 (20%) that serves young adults.

In bivariate analyses, there was an association of clinical signs, including cervical discharge, cervicitis, and cervical motion tenderness, with *C*. *trachomatis* infection but these did not correlate with symptoms. As expected, the majority of women with *C*. *trachomatis* were asymptomatic. Of the 103 women with *C*. *trachomatis*, only 30 sought an evaluation for infertility, lower abdominal and/or other symptoms, or a desire to be tested for STIs. Furthermore, only 11 (10.7%) met the criteria for treatment based on syndromic management. The WHO developed this approach using a combination of symptoms and signs that could be easily recognized by health care providers to guide decisions regarding empiric treatment for *C*. *trachomatis*, *N*. *gonorrhoeae* and other STIs [12]. Although the bivariate findings did not hold up in multivariate analyses, the data suggest that syndromic management is failing to identify a substantial number of women with *C*. *trachomatis* infections. Our findings are consistent with a recent study of adolescents and young adults in South Africa where symptom-based reporting of a potential genital tract infection used for syndromic management had a sensitivity of only 14% compared to laboratory testing [35]. In our study, the sensitivity was 10.7% (11/103). The poor sensitivity and specificity of this approach has likely led to the under treatment of *C*. *trachomatis* STIs and a large reservoir of individuals in Fiji who can not only transmit *C*. *trachomatis* to their sexual partners but develop upper genital tract sequelae, including infertility. Further, *C*. *trachomatis* is a risk factor for HIV acquisition [14]. While HIV prevalence remains at approximately 0.1% in the PICT [36, 37], except for Papua New Guinea, the epidemic proportions of *C*. *trachomatis* with an economy built on tourism leaves Fiji and other PICT extremely vulnerable to the rapid spread of HIV and its consequent morbidity and mortality.

Over treatment of women with and without STIs remains an important public health concern that can promote antibiotic resistance. While there are reports of drug resistance to *C*. *trachomatis* [38], the consensus in the field is that resistance to azithromycin and doxycycline, the primary antibiotics used to treat *C*. *trachomatis*, is rare [39]. This is not the case for *N*. *gonorrhoeae* where antibiotic resistance has become a major problem for infection control. Reports of strains expressing high-level resistance to all extended-spectrum cephalosporins along with other antibiotics [40] have made these ‘superbugs’ an urgent global public health threat. In a recent study of women tested for *C*. *trachomatis* and *N*. *gonorrhoeae* in U.S. emergency departments, 46.5% and 46.7%, respectively, of uninfected women were treated [41]. In Fiji, 123 (26%) of 474 uninfected women in our study were treated. These high rates of treatment are a poor use of limited resources and further fuel the global epidemic of drug resistant bacteria, especially *N*. *gonorrhoeae*, in the PICT.

Although women from seven MoHMS Health Centers and five urban and rural outreach clinics participated in the study, one limitation is that consecutive enrollment may not represent all populations in Fiji. This is suggested by the higher number of university-level educated and married women in the study that may underestimate the overall prevalence of *C*. *trachomatis*, indicating an even greater need for future research and action in Fiji. Overall, however, the participants were representative of the other demographics of the Fijian population. Further, it was beyond the scope of the current study to pursue partner notification and testing, although each woman who tested positive for *C*. *trachomatis* was advised of the importance of having her partner(s) evaluated.

Our study demonstrates that genital *C*. *trachomatis* rates are hyperendemic among non-pregnant women and are similar to documented rates among antenatal women in Fiji for those under 25 years of age from over a decade ago (30.5% vs 34%) [5]. The high prevalence of infection and lack of condom use has likely contributed to the growing epidemic, and indicate that *C*. *trachomatis* is a primary factor for infertility among Fijians who have some of the highest rates in the world: ≥3% for primary infertility and ≥13% for secondary infertility [42]. Syndromic management is ineffective because the majority of infected women in our study did not have symptoms and were not actively seeking an evaluation for infertility or screening for STIs. Of the *C*. *trachomatis* positive participants, 92 (89%) would have been mis-diagnosed and not treated.

While our cross-sectional study was focused on the Fijian population, we believe that our findings will be relevant to other populations in other PICT including the U.S. territories of Guam, Commonwealth of the Northern Mariana Islands, and American Samoa, in addition to the U.S. state of Hawaii where the population is primarily composed of Pacific Islanders similar to Fiji. Importantly, of all the populations in the PICT, Fijians have the highest representation in the U.S. and, along with Hawaiian and other Pacific Islanders, are designated by NIH as health disparity populations [43, 44].

Rapid, sensitive and inexpensive point-of-care diagnostics for *C*. *trachomatis* are desperately needed but not yet available. Therefore, targeted interventions that include education and screening of at-risk adolescent and young adult women and their partners along with appropriate treatment would advance *C*. *trachomatis* infection control and dramatically decrease transmission and the consequent sequelae of these infections in Fiji and the PICT.

## Supporting information

S1 Checklist STROBE checklist. (DOC)
